# Brain MR image denoising for Rician noise using pre-smooth non-local means filter

**DOI:** 10.1186/1475-925X-14-2

**Published:** 2015-01-09

**Authors:** Jian Yang, Jingfan Fan, Danni Ai, Shoujun Zhou, Songyuan Tang, Yongtian Wang

**Affiliations:** Beijing Engineering Research Center of Mixed Reality and Advanced Display, School of Optics and Electronics, Beijing Institute of Technology, Beijing, 10081 China; Shenzhen Institutes of Advanced Technology, Chinese Academy of Sciences, Shenzhen, 518055 China

## Abstract

**Background:**

Magnetic resonance imaging (MRI) is corrupted by Rician noise, which is image dependent and computed from both real and imaginary images. Rician noise makes image-based quantitative measurement difficult. The non-local means (NLM) filter has been proven to be effective against additive noise.

**Methods:**

Considering the characteristics of both Rician noise and the NLM filter, this study proposes a frame for a pre-smoothing NLM (PSNLM) filter combined with image transformation. In the PSNLM frame, noisy MRI is first transformed into an image in which noise can be treated as additive noise. Second, the transformed MRI is pre-smoothed via a traditional denoising method. Third, the NLM filter is applied to the transformed MRI, with weights that are computed from the pre-smoothed image. Finally, inverse transformation is performed on the denoised MRI to obtain the denoising results.

**Results:**

To test the performance of the proposed method, both simulated and real patient data are used, and various pre-smoothing (Gaussian, median, and anisotropic filters) and image transformation [squared magnitude of the MRI, and forward and inverse variance-stabilizing trans-formations (VST)] methods are used to reduce noise. The performance of the proposed method is evaluated through visual inspection and quantitative comparison of the peak signal-to-noise ratio of the simulated data. The real data include Alzheimer’s disease patients and normal controls. For the real patient data, the performance of the proposed method is evaluated by detecting atrophy regions in the hippocampus and the parahippocampal gyrus.

**Conclusions:**

The comparison of the experimental results demonstrates that using a Gaussian pre-smoothing filter and VST produce the best results for the peak signal-to-noise ratio (PSNR) and atrophy detection.

## Background

Magnetic resonance imaging (MRI) images of the brain have an important role in diagnosing many neurological diseases, such as Parkinson’s disease, Alzheimer’s disease (AD), brain tumors, and stroke. Analyzing MRI images can help surgeons make appropriate decisions. However, MRI noises degrade image quality, which negatively affects image processing and analysis works, such as registration, segmentation, classification, and visualization. To obtain reliable analysis results, removing MRI image noises is necessary before further image processing can be conducted.

The technique of removing noises from images is called “image denoising,” which is an important image pre-processing step. Although many image denoising methods have been developed, denoising remains a challenge because these methods produce artifacts and blurry images [1]. Most denoising methods still cannot provide desirable results [2].

The most commonly used noise model is the additive noise model, that is,
1

where noise n is independent and Gaussian distribution with zero-mean and known standard deviation, *I*_0_ is the true signal, and I is the observed signal. Most denoising methods have been developed by using the additive noise model. These methods are classified into two major categories: spatial filtering and transform domain filtering methods.

Spatial domain techniques directly deal with image pixels. A spatial image filter is an image operation wherein each pixel or voxel value I(u) is transformed through a function of the intensities of the pixels or voxels within a neighborhood (u). Traditional spatial image filters include Gaussian [3], median [4], Wiener [5], diffusion [6], and bilateral filters. Gaussian and median filters remove noise in a small constant region and blur images. An anisotropic diffusion filter preserves the edges of images, but erases small features and generates a mask effect in uniform regions of the denoised images. These denoising methods significantly remove noise but cause blurred images and add artifacts to the images. A transform domain image filter transforms images from the space domain into another domain, such as the frequency and wavelet domains [7], and then processes images in the new domain. The wavelet thresholding method can significantly reduce noise, but introduces characteristic artifacts. In this study, we mainly focus on the spatial domain filter.

A non-local mean (NLM) algorithm has been proposed recently [8]. This algorithm provides good edge-preserving results. Each pixel of the denoised image from the NLM algorithm can represent the weighted average of all pixels in the noisy image by using a Gaussian function as the smoothing function. The NLM filter has been proven to be an effective denoising method, particularly against additive noise.

Many MRI denoising methods have been reported. Gaussian filter smoothing is a key step in voxel-based morphometry (VBM) analysis [9]. A Wiener filter uses its neighborhood to estimate its parameters [10]. An anisotropic filter combines local linear minimum mean squared error (MSE) filters to remove MRI noise [11]. A trilinear filter achieves edge-preserving results by integrating geometric, photometric, and local structural similarities [12]. Noise estimation methods in the wavelet domain are also used in MRI denoising. MRI in the wavelet domain is decomposed into sub-bands at various scales. Coefficients are processed with soft or hard thresholding to estimate signal components [13]. The NLM filter is also used in MRI denoising [14, 15].

The noise in the MRI images is modeled according to the coil number in the imaging system. In the single coil system, the noisy distribution in MRI images can be modeled as a Rician distribution [16], which assumes that the real part and imaginary part of the MRI image is an uncorrelated Gaussian distribution with a zero mean and equal variance. While in the multi-coil system (parallel MRI), the magnitude of noises follows a non-central Chi distribution [17] with a sum-of-squares (SoS) reconstruction. The noise in the MRI images acquired from the generalized auto calibrating partially parallel acquisition (GRAPPA) reconstruction [18] can be modeled by the non-central Chi distribution. Actually, the Rician distribution is a special case of the nonCentral Chi distribution. To apply the additive noise model, the nonCentral Chi distribution should be transformed into the Gaussian distribution in the transformed space. Therefore, the above-mentioned denoising methods can be applied to the transformed data. After inverse transformation of the denoising data, the noise is removed from the MRI images. Many mthods have been proposed to deal with the problem, such as the local moment distribution based method [19], nonlocal maximum likelihood (NLML) estimation [20], and the effective variance of noise from the composite magnitude signal of MR data [20].

In this study, we focus on the single coil system which produces noises with Rician distribution. To remove noise and apply the additive noise model, Rician noise should be transformed into an independent Gaussian noise. The squared magnitude correction is widely used for MRI denoising [13]. The standard deviation of noise is estimated from a dark uniform background. Recently, a forward and inverse variance-stabilizing transformation (VST) [21] has been proposed for the Rician distribution. The forward VST removes the dependency of the noise variance on the observed image, and the inverse VST compensates the bias in the filtered image.

A frame for a pre-smoothing NLM (PSNLM) filter is proposed in the study. MRI noise is first transformed into additive noise and then smoothed by a traditional denoising filter (Gaussian, median, or anisotropic). Next, the weight of the NLM algorithm is calculated from the smooth image, and noise is removed by the NLM filter. Finally, inverse transformation is used to obtain accurate results. Squared magnitude transformations, as well as forward and inverse variance-stabilizing transformations (VST) [21], are adopted to transform noise in the proposed method.

## Methods

**Figure 1:**
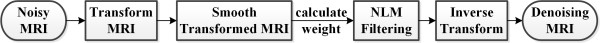
**Block diagram of the proposed method.**

The proposed method includes four steps: (1) transformation of noisy MRI, (2) pre-smoothing of the transformed MRI, (3) noise removal by the NLM filter, and (4) unbiased correction (inverse transformation) of the denoised image. The block diagram is shown in Figure 1.

### MRI Model

MRI images are widely used in medical applications because they prevent the formation of phase artifacts by discarding phase information. MRI is computed from both real and imaginary images, which are assumed to contain Gaussian distribution noises with zero means [14]. Thus, noise is image dependent, which follows a Rician distribution, and makes removing noises difficult. A complex MRI is represented as follows:
2

where z is the complex MRI; z_*real*_ and z_*imaginary*_ are the real and imaginary components, respectively, which are independently corrupted by Gaussian white noises n_1_ and n_2_, respectively, with zero mean and standard deviation *σ*; and r and θ are the magnitude and phase of the original MRI, respectively.

A measured noisy MRI can be represented as follows:
3

The measured MRI magnitude |*z*| is under Rician distribution and is represented as follows:
4

where *I*_0_ denotes the modified Bessel function of order zero.

Estimating noise from the measured magnitude |*z*| of MRI is difficult. The most commonly used method is to square the magnitude of MRI. Recently, VST is proposed for Rician noise processing.

### The squared magnitude method

Based on Eq. (2), the expectation of |*z*|^2^ is given as follows:
5

This equation indicates that the squared magnitude MRI has a 2*σ*^2^ noise bias and is image independent. For an original brain MRI, the background is an empty region with zero intensity. Thus, *σ* in the background region can be estimated as follows:
6

where *μ* is the mean intensity of the squared magnitude MRI in the background region. |*z*|^2^ can be filtered by using any noise removal method Φ. According to Eq. (4), the unbiased *I*_*u*_ is estimated as follows:
7

### Background of the brain

The background of the brain can be estimated by using any segmentation method. In this study, the Otsu method [22], which is an automatic image segmentation technique, is adopted to extract the background region. This algorithm assumes that two classes of pixels or bi-modal histogram (e.g., foreground and background) are found in the image and exhaustively searches for the threshold that maximizes inter-class variance [22, 23]. The process includes median filtering, Otsu segmentation, morphological close operation, and filling the holes in the image.

**Figure 2:**
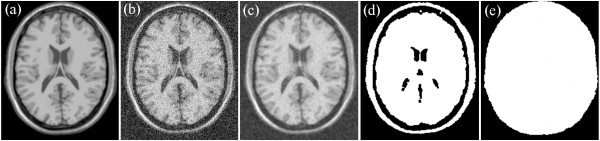
**An example of extracting background from T1-weighted MRI. (a)** the original T1 weight MRI, **(b)** the MRI with Rician noise, **(c)** the result of the median filtering, **(d)** the result of the morphological close operation, and **(e)** the result of the brain region.

**Figure 3:**
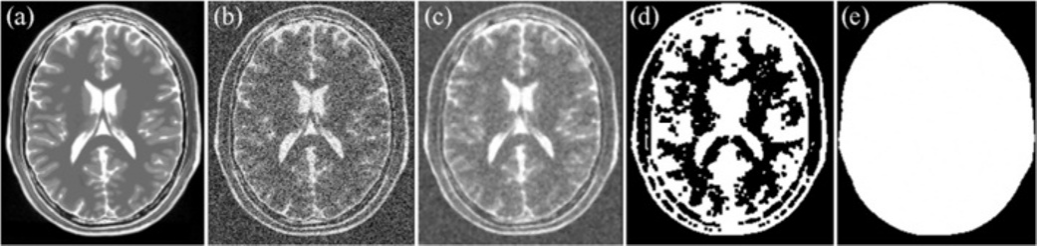
**An example of extracting background from T2-weighted MRI. (a)** the original T2 weight MRI, **(b)** the MRI with Rician noise, **(c)** the result of the median filtering, **(d)** the result of the morphological close operation, and **(e)** the result of the brain region.

**Figure 4:**
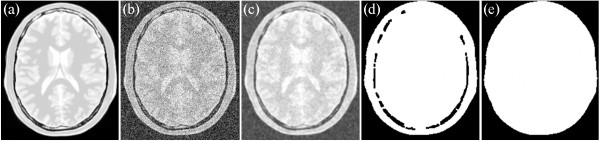
**An example of extracting background from PD-weighted MRI. (a)** the original PD weight MRI, **(b)** the MRI with Rician noise, **(c)** the result of the median filtering, **(d)** the result of the morphological close operation, and **(e)** the result of the brain region.

If the skulls of T1, T2, and PD are clear, then the mask of the brain is easily segmented as well as the background that uses the process. An example is shown in Figures 2, 3 and 4. Figure 2(a) shows the original T1-weighted MRI; Figure 2(b) shows the T1-weighted MRI with Rician noise; Figure 2(c) presents the result of median filtering; Figure 2(d) provides the result of the morphological close operation; and Figure 2(e) presents the result after the holes are filled. Figure 3(a) shows the original T2-weighted MRI; Figure 3(b) shows the T2-weighted MRI with Rician noise; Figure 3(c) presents the result of median filtering; Figure 3(d) provides the result of the morphological close operation; and Figure 3(e) presents the result after the holes are filled. Figure 4(a) shows the original PD-weighted MRI; Figure 4(b) shows the PD-weighted MRI with Rician noise; Figure 4(c) presents the result of median filtering; Figure 4(d) provides the result of the morphological close operation; and Figure 4(e) presents the result after the holes are filled. In these figures, the bright area is the brain region, whereas the dark area is the background.

**Figure 5:**
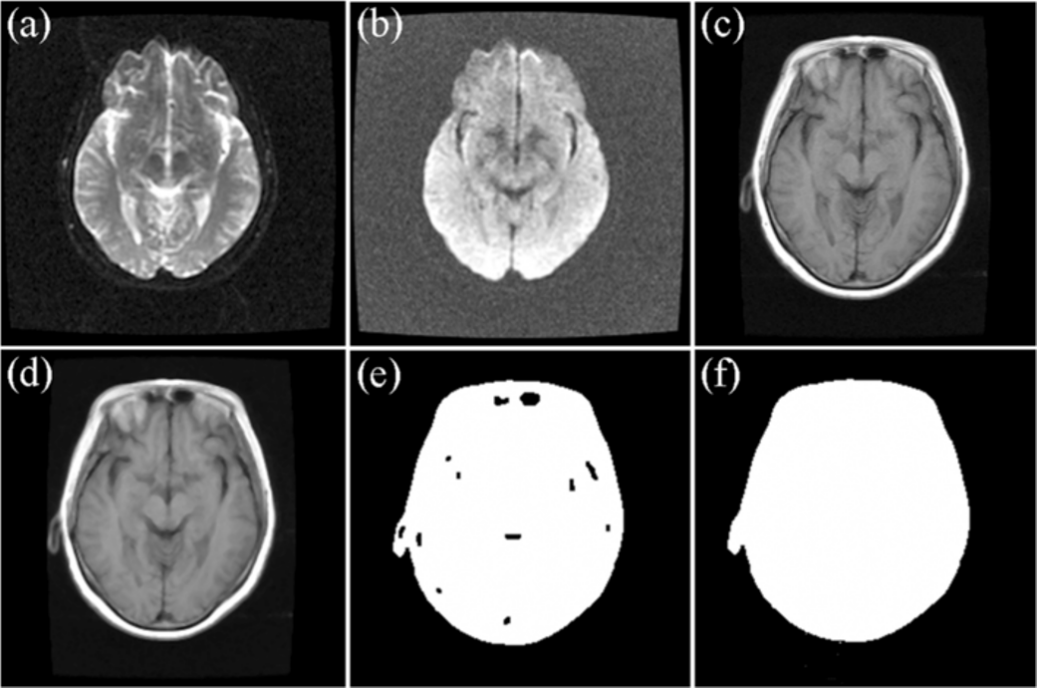
**Images of a patient with meningiomais, T2- and PD- weighted MRI are registered to T1 weighted MRI, which is clear and easy to extract background.** The background region of T1 weighted MRI is also the background region of registered T2- and PD- weighted MRI. **(a)** T2 weight MRI, **(b)** PD weight MRI, and **(c)** T1 weight MRI, **(d)** the result of the median filtering for T1 weight MRI, **(e)** the result of the corresponding morphological close operation, and **(f)** the final result of the background (dark region).

In many cases, the skull is clear only in the T1-weighted MRI, and its intensities in the T2- and PD-weighted MRIs are close to the background. Figure 5 shows the T1-, T2-, and PD-weighted MRIs of a patient with meningioma. Figure 5(a) shows the T2-weighted MRI; Figure 5(b) shows the PD-weighted MRI; and Figure 5(c) shows the T1-weighted MRI. In such situations, the brain region and the background are easily segmented for the T1-weighted image, as shown in Figure 5(d) to (f). Figure 5(d) presents the result of median filtering; Figure 5(e) provides the result of the morphological close operation; and Figure 5(f) presents the final result. To obtain the background of the corresponding T2- and PD-weighted MRIs, we align the T1-weighted MRI to the T2-and PD-weighted MRIs, and transform the background of the T1-weighted MRI into the T2-and PD-weighted spaces. The transformed background can be used as the background of the T2- and PD-weighted MRIs. Given that the images belong to the same person, aligning them is easy. The images are multi-modal; therefore, a rigid registration with mutual information is selected to align the images [24].

### The method of variance-stabilization

This method involves the optimal forward and inverse VSTs for Rician noise. Forward transformations (f) treat MRI noise as an additive noise with a unitary variance. Thus, noise can be removed by using the NLM filter. Denoising MRI can be achieved through inverse transformation (I).

Forward transformations can be represented as follows:
8

where a is a constant . The details can be found in [16]. In this study, *σ* is obtained from the background through Eq. (5), as described in the above section.

A denoising method Φ is then applied to *f*(*z*) to obtain a noise removal image D.
9

Inverse transformation is then applied to D to obtain unbiased estimation *I*_*u*_ as follows:
10

### NLM filter

For a 3D image measuring *N*_*x*_ × *N*_*y*_ × *N*_*z*_ in the NLM filter, the estimated intensity NLM(|*z*|(*i*, *j*, *k*)) of voxel (i,j,k) is the weighted average of all voxel intensities in the noisy image |*z*|, which is defined as follows:
11

where NLM(|*z*|(*i*, *j*, *k*)) is the intensity of the denoised image at position (i,j,k) through the NLM filter, |*z*|(*l*, *m*, *n*) is the intensity of the noisy image at (i,j,k), and w(*i*, *j*, *k*, *l*, *m*, *n*) is the weighting coefficient that is defined as follows:
12

where *h* is the filtering degree. *N*_*i*,*j*,*k*_ and *N*_*l*,*m*,*n*_ are the blocks centered at voxels (i,j,k) and (l,m,n), respectively, with both blocks measuring *n*_*x*_ × *n*_*y*_ × *n*_*z*_; G_*a*_ is a Gaussian weighting function with zero mean and standard deviation, where a is typically set to one; and Z(*i*, *j*, *k*) is a normalizing constant that is defined as follows:
13

According to the Eq. (10)-(12), to modify each noisy voxel |*z*|(*i*, *j*, *k*), its neighborhood |*z*|(N_*i*,*j*,*k*_) is compared with other noisy voxel’s neighborhood |*z*|(N_*l*,*m*,*n*_) in the entire image. It needs huge computation. Considering the computational efficiency, usually the search window is selected to be a much smaller than the size of the entire image and is *s*_*x*_ × *s*_*y*_ × *s*_*z*_.

### The Pre-smooth Non-local Means Filter (PSNLM)

Before using the NLM filter, MRI |z| is first transformed into *I*_*t*_ via forward transformation f (squared magnitude or variance stabilization). Subsequently, *I*_*t*_ is smoothed by a traditional filter S (Gaussian, median, or anisotropic) to become *I*_*s*_ . When the NLM filter is used to remove noise, the weight and normalizing constant are calculated from *I*_*s*_ as follows:
141516

An unbiased estimation is obtained through inverse transformation of the PSNLM result. PSNLM1 and PSNLM2 are used to represent squared magnitude and VST, respectively.
1718

The summary of PSNLM is listed in Table 1.

**Table 1:** **Summary of the PSENLM**

PSENLM1	PSENLM2
(1) Extract background	(1) Transform noisy image by the forward variance-stabilization transformation
(2) Compute the noise variance *σ* ^2^ according Eq. (5)	(2) Smooth the transformed image
(3) Compute the squared magitude of noisy image	(3) Compute the weigh term from the smoothed image
(4) Smooth the squared magitude of noisy image	(4) Obtained final result by the inverse variance-stabilization transforming of the NLM denoising result
(5) Compute the weigh term from the smoothed image	
(6) Correct bias by subtracting 2*σ* ^2^ from the NLM denoising result. The final result is obtained by squared root of the bias correction	

### Evaluation

For the simulated data, the results are measured quantitatively by the peak signal-to-noise ratio (PSNR), which is a commonly used objective metrics, and visual inspection. PSNR is defined as follows [25]:
19

where *C* is the maximum intensity in which the image can be given. MSE between the final denoised image and the original image is defined as follows:
2021

where *PSNLM*1(*i*, *j*, *k*) and *PSNLM*2(*i*, *j*, *k*) are the voxels of PSNLM1 and PSNLM2, respectively, at position (*i*, *j*, *k*); and *r*(*i*, *j*, *k*) is the original MRI at position (*i*, *j*, *k*). A large PSNR indicates good results.

For real patient data, the performance of the proposed method in detecting brain atrophy among patients with AD is evaluated by using VBM [9, 26]. In VBM studies, all individual brain images are spatially normalized onto template space through a registration algorithm. Based on the estimated deformation field, each brain morphometry information can be measured by using a Jacobian map, which reflects regional volumetric changes that are compared with the template image. Subsequently, voxel-wise group analysis is performed by using SPM software to detect brain atrophy [27].

### Experiment data

The experiment data include both simulated and real patient data. The 3D-simulated MRI images that are used in the experiments are downloaded from the BrainWeb database [2]. The simulated T1-, T2-, and PD-weighted MRI images without noise are downloaded. The size of each image is 181 × 217 × 181 and the intensity is 256 bins. Rician noise is simulated according to Eq. (2), with *n*_1_ ~ N(0, σ), *n*_2_ ~ N(0, σ), θ = 0, and r is a an image without noise, which can be taken as the ground truth. Various levels of noise (percent %) are added to the three MRI images.
22

where t is the intensity of the brightest tissue, which is 150, 250, and 255 for T1-, T2-, and PD-weighted MRI images, respectively, with 256 bins. The noisy image is given as follows:
23

In this case, randn(size(r)) produces data with the same size as the ground truth image, and the mean and standard deviation of the data are 0 and 1, respectively. The real patient data that are used in this study are downloaded from the Alzheimer’s Disease Neuroimaging Initiative (ADNI) database [27], which includes many T1-weighted MRI images of AD patients and normal controls (NC). To test the performance of the proposed method, 20 NCs and 20 AD patients with T1-weighted MRI images of the baseline are randomly selected from the database.

## Results and discussion

The experimental results are obtained from both simulated and real patient data.

### Simulated data

**Figure 6:**
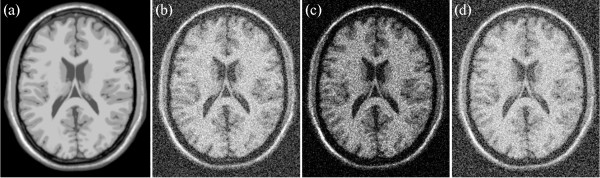
**An example of T1 image: (a) original T1 weighted MRI, (b) 17% noisy T1 weighted MRI, (c) squared magnitude of the noisy T1 weighted MRI, (d) the forward variance-stabilization transformation of the noisy T1 weighted MRI.**

**Figure 7:**
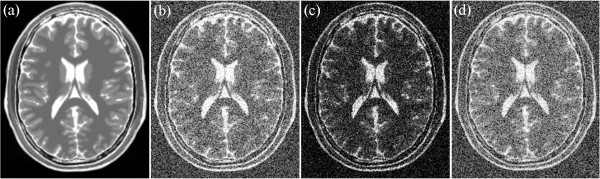
**An example of T2 image: (a) original T2 weighted MRI, (b) 17% noisy T2 weighted MRI, (c) squared magnitude of the noisy T2 weighted MRI, (d) the forward variance-stabilization transformation of the noisy T1 weighted MRI.**

**Figure 8:**
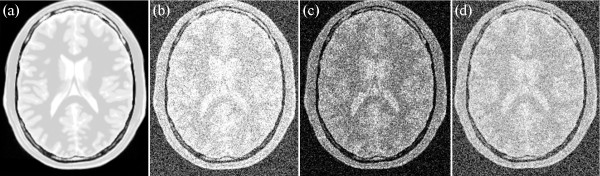
**An example of PD image: (a) original PD weighted MRI, (b) 17% noisy PD weighted MRI, (c) squared magnitude of the noisy PD weighted MRI, (d) the forward variance-stabilization transformation of the noisy PD weighted MRI.**

For the simulated data, several noise levels, namely, 9%, 13%, 17%, and 21% are added into T1-, T2-, and PD-weighted MRI images. Examples of T1-, T2-, and PD-weighted MRI images with 17% noise are shown in Figures 6, 7 and 8, respectively. Figure 6(a) shows the original T1-weighted MRI image, whereas Figure 6(b) shows the corresponding noisy MRI image. Figure 7(a) shows the original T2-weighted MRI image, whereas Figure 7(b) shows the corresponding noisy T2-weighted MRI image. Figure 8(a) shows the original PD-weighted MRI image, whereas Figure 8(b) shows the corresponding noisy MRI image. In this study, squared magnitude and VST are used to transform noisy MRI image into additive noise. Figure 6(c) shows the squared magnitude noisy T1-weighted MRI image, whereas Figure 6(d) shows the forward VST of the noisy T1-weighted MRI image. Figure 6(c) shows the squared magnitude noisy T2-weighted MRI image, whereas Figure 7(d) shows the forward VST of the noisy T2-weighted MRI image. Figure 8(c) shows the squared magnitude noisy PD-weighted MRI image, whereas Figure 8(d) shows the forward VST of the noisy PD-weighted MRI image.

Moreover, several filters are used to pre-smooth images. In this study, common filters, such as Gaussian, median, and anisotropic filters, are used to pre-smooth MRI images. The NLM algorithm has three parameters: patch size, search window size *s*_*x*_ × *s*_*y*_ × *s*_*z*_, and filtering degree *h*. In many NLM-based MRI denoising cases, patch size is set as 5 × 5 × 5, and search window size is set as 11 × 11 × 11 for MRI images with a voxel size of 1 × 1 × 1 *mm*^3^ [13]. Thus, the same parameters are used in this study. h is typically set as proportional to the noise level of the image. In our experiments, different h values are tested, and we selected the h value that produces the largest PSNR. For comparison, the PSNR of different methods are calculated. These methods include the original NLM algorithm, unbiased correction (UNLM) by the squared magnitude, as well as VST, PSNLM1, and PSNLM2 with Gaussian, median, and anisotrpic filters, respectively.

### Comparison of PSNR

**Figure 9:**
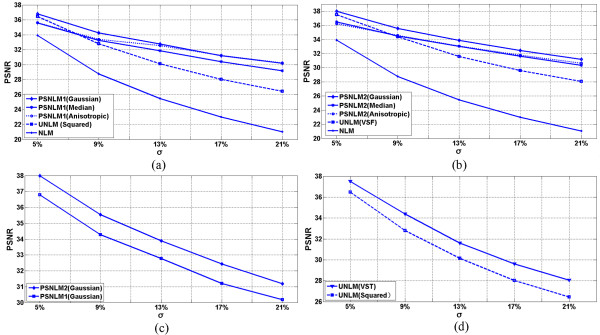
**T1 weighted MRI: (a) PSNR of the original NLM, UNLM with squared magnitude, and PSNLM1 with Gaussian, median, and anisotropic filters, (b) PSNR of the original NLM, UNLM with VST, and PSNLM2 with Gaussian, median, and anisotropic filters. (c)** PSNR of PSNLM with Gaussian filter, and **(d)** PSNR of ULM with VST and squared magnitude.

**Figure 10:**
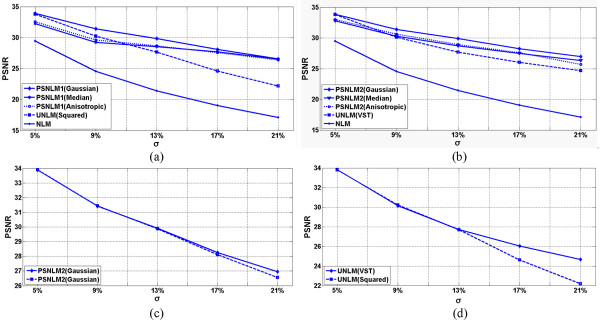
**PD weighted MRI, (a) PSNR of the original NLM, UNLM with squared magnitude, and PSNLM1 with Gaussian, median, and anisotropic filters, (b) PSNR of the original NLM, UNLM with VST, and PSNLM2 with Gaussian, median, and anisotropic filters. (c)** PSNR of PSNLM with Gaussian filter, and **(d)** PSNR of ULM with VST and squared magnitude.

**Figure 11:**
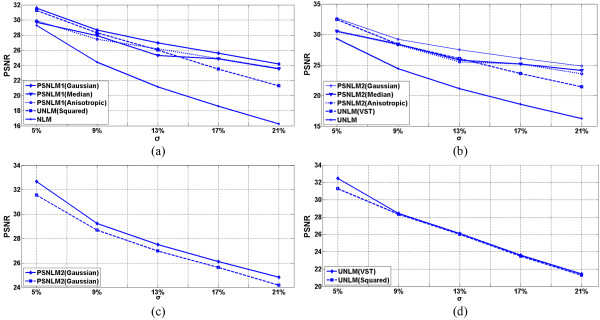
**T2 weighted MRI, (a) PSNR of the original NLM, UNLM with squared magnitude, and PSNLM1 with Gaussian, median, and anisotropic filters, (b) PSNR of the original NLM, UNLM with VST, and PSNLM2 with Gaussian, median, and anisotropic filters. (c)** PSNR of PSNLM with Gaussian filter, and **(d)** PSNR of ULM with VST and squared magnitude.

Figures 9, 10 and 11 show the comparisons of the experiment results with PSNR. Figure 9 shows the PSNR of the T1-weighted MRI image at different noise levels. Figure 9(a) includes the PSNR of the original NLM, UNLM with squared magnitude, and PSNLM1 with Gaussian, median, and anisotropic filters. The curve of the PSNLM1 with a Gaussian filter is at the top. Figure 9(b) includes the PSNR of the original NLM, UNLM with VST, and PSNLM2 with Gaussian, median, and anisotropic filters. The curve of the PSNLM2 with a Gaussian filter is also at the top. The PSNR of the original NLM algorithm are at the bottom of both figures. The PSNR curves of the UNLM and PSNLM with median and anisotropic filters are between that of the PSNLM with a Gaussian filter and NLM. The proposed method can improve the denoising results. Pre-smoothing with a Gaussian filter achieves the best results. Figure 9(c) shows the PSNR curves of the PSNLM2 and PSNLM1with a Gaussian filter. The PSNR of PSNLM2 is larger than that of PSNLM1. This result indicates that the proposed method with VST and a Gaussian filter performs the best. Figure 9(d) shows that the PSNR of the UNLM with VST is larger than that of the UNLM with the squared magnitude. Thus, VST is the better choice between the two.

Figures 10 and 11 show the PSNR of the PD- and T2-weighted MRI images with different noise levels. The results are similar to that of the T1-weighted MRI image. The PSNR curves with a Gaussian filter are at the top, as shown in Figure 10(a) and (b) for the PD-weighted MRI images and Figure 11(a) and (b) for the T2-weighted MRI images. Figures 10(c) and 11(c) show the PSNR of the PSNLM1 and PSNLM2 with a Gaussian filter for the PD- and T2-weighted MRI images. The two curves are extremely close in Figure 10(c). The PSNR of PSNLM2 is slightly larger than that of PSNLM1. In Figure 11(c), the PSNR of PSNLM2 is larger than that of PSNLM1. Figures 10(d) and 11(d) show the PSNR of the UNLM with VST and with the squared magnitude, respectively. The curves are extremely close at a low noise level for the PD-weighted MRI image [Figure 10(d)] and at a high noise level for the T2-weighted MRI image.

### Comparison by visual inspection

**Figure 12:**
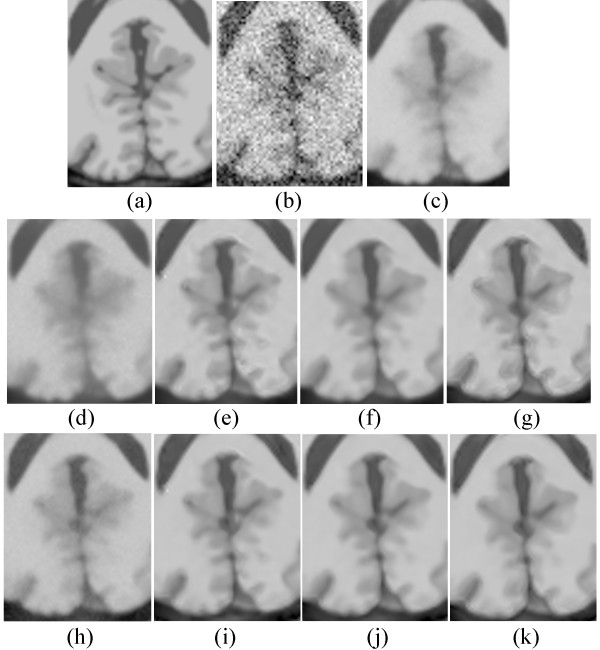
**Comparison of experiment results on T1-weighted MRI. (a)** original MRI, **(b)** noisy MRI with 17% Rician noise, **(c)** results of the original NLM filter, **(d)** results of the UNLM with squared magnitude transformation, **(e)** results of the PSNLM1 with median filter, **(f)** results of the PSNLM1 with Gaussian filter, **(g)** results of the PSNLM1 with anisotropic filter, **(h)** results of the UNLM with VST, **(i)** results of the PSNLM2 with median filter, **(j)** results of the PSNLM2 with Gaussian filter, **(k)** results of the PSNLM2 with anisotropic filter.

**Figure 13:**
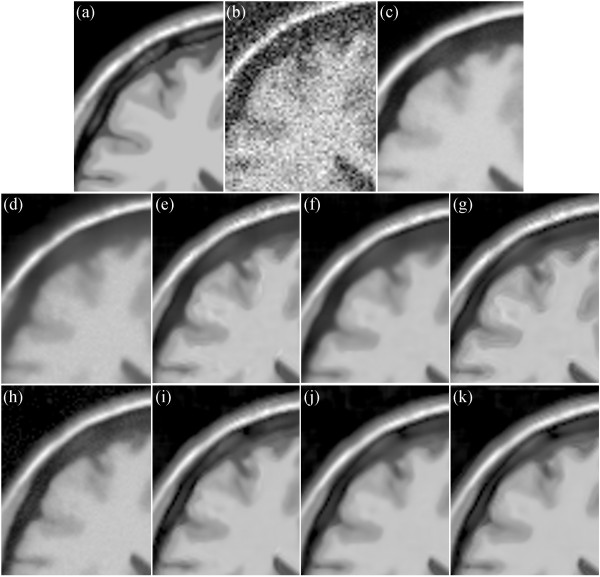
**Comparison of experiment results on T1-weighted MRI. (a)** original MRI, **(b)** noisy MRI with 17% Rician noise, **(c)** results of the original NLM filter, **(d)** results of the UNLM with squared magnitude transformation, **(e)** results of the PSNLM1 with median filter, **(f)** results of the PSNLM1 with Gaussian filter, **(g)** results of the PSNLM1 with anisotropic filter, **(h)** results of the UNLM with VST, **(i)** results of the PSNLM2 with median filter, **(j)** results of the PSNLM2 with Gaussian filter, **(k)** results of the PSNLM2 with anisotropic filter.

Figure 12 shows the enlarged regions of the experimental results on the T1-weighted MRI image. Figure 12(a) shows an enlarged region of the original MRI, and Figure 12(b) shows noisy MRI with 17% Rician noise. Figure 12(c) presents the result of the original NLM. The edges are obviously blurred. Figure 12(d) and (h) present the results of the UNLM with the squared magnitude and VST, respectively. Figure 12(h) is clearer than Figure 12(d). Although both results are better than that of the original NLM algorithm, the images are still blurred. Figure 12(e) to (g) present the results of the PSNLM1 with median, Gaussian, and anisotropic filters, respectively, whereas Figure 12(i) to (k) present the results of the PSNLM2 with median, Gaussian, and anisotropic filters, respectively. All results are significantly improved compared with Figure 12(d) and (h). However, some artifacts are observed in Figure 12(e),(g),(i), and (k). Figure 12(f) and (j) show that the results are extremely close to and similar to those of the original image. Nevertheless, the result in Figure 12(j) is slightly better than those in the other figures. Figure 13 is another enlarged region of the experimental results on the T1-weighted MRI image. Comparably, there are some artifacts in Figure 13(e),(f),(g) and (i), Figure 13(c),(d) and (h) are slight blurred, while Figure 13(j) and (k) are clearer than the others. It is obvious that the results of PSNLM2 with Gaussian and median filters are better than the other methods.

**Figure 14:**
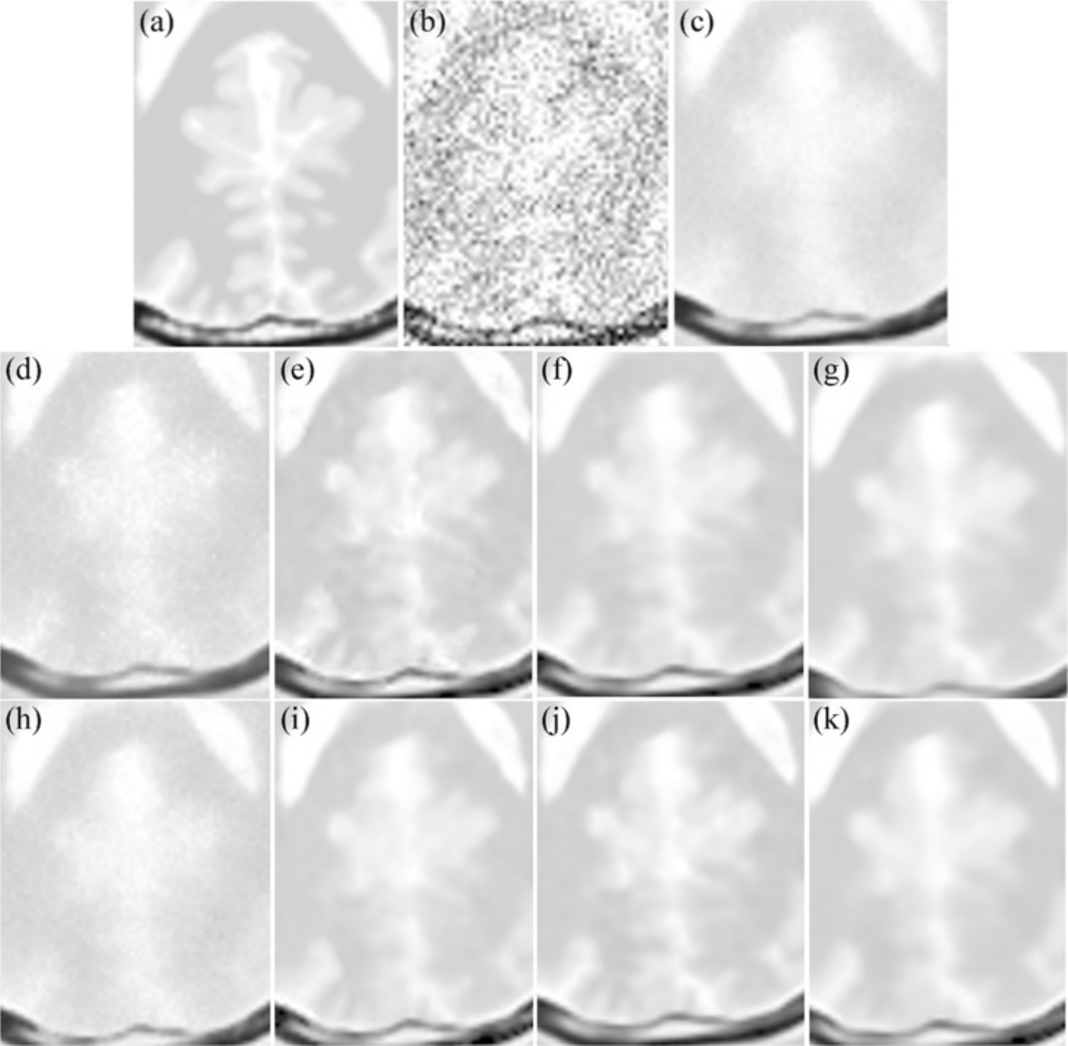
**Comparison of experiment results on PD-weighted MRI. (a)** original MRI, **(b)** noisy MRI with 17% Rician noise, **(c)** results of the original NLM filter, **(d)** results of the UNLM with squared magnitude transformation, **(e)** results of the PSNLM1 with median filter, **(f)** results of the PSNLM1 with Gaussian filter, **(g)** results of the PSNLM1 with anisotropic filter, **(h)** results of the UNLM with VST, **(i)** results of the PSNLM2 with median filter, **(j)** results of the PSNLM2 with Gaussian filter, **(k)** results of the PSNLM2 with anisotropic filter.

Figure 14 presents the results on the PD-weighted MRI image with 17% Rician noise. Figure 14(a) shows an enlarged region of the original MRI image, and Figure 14(b) shows the noisy MRI image. Figure 14(c) presents the result of the original NLM. Figure 14(d) and (h) present the results of the UNLM with the squared magnitude and VST, respectively. Figure 14(e) to (g) present the results of the PSNLM1 with median, Gaussian, and anisotropic filters, respectively. Figure 14(i) to (k) present the results of the PSNLM2 with median, Gaussian, and anisotropic filters, respectively. It is obvious that Figure 14(c), (d) and (h) are blurred, while the pre-smooth technique significantly improves the result, as can be seen in Figure 14(j).

**Figure 15:**
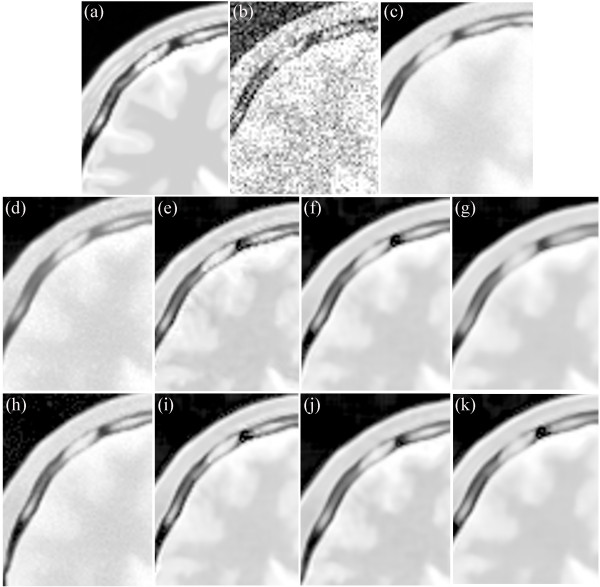
**Comparison of experiment results on PD-weighted MRI. (a)** original MRI, **(b)** noisy MRI with 17% Rician noise, **(c)** results of the original NLM filter, **(d)** results of the UNLM with squared magnitude transformation, **(e)** results of the PSNLM1 with median filter, **(f)** results of the PSNLM1 with Gaussian filter, **(g)** results of the PSNLM1 with anisotropic filter, **(h)** results of the UNLM with VST, **(i)** results of the PSNLM2 with median filter, **(j)** results of the PSNLM2 with Gaussian filter, **(k)** results of the PSNLM2 with anisotropic filter.

Figure 15 is another enlarged region of the experiment results on the PD-weighted MRI image. It is obvious that Figure 15(c),(d) and (h) are, to some extent, blurred. While the proposed pre-smooth technique greatly improves the result, as can be seen in Figure 15(j).

**Figure 16:**
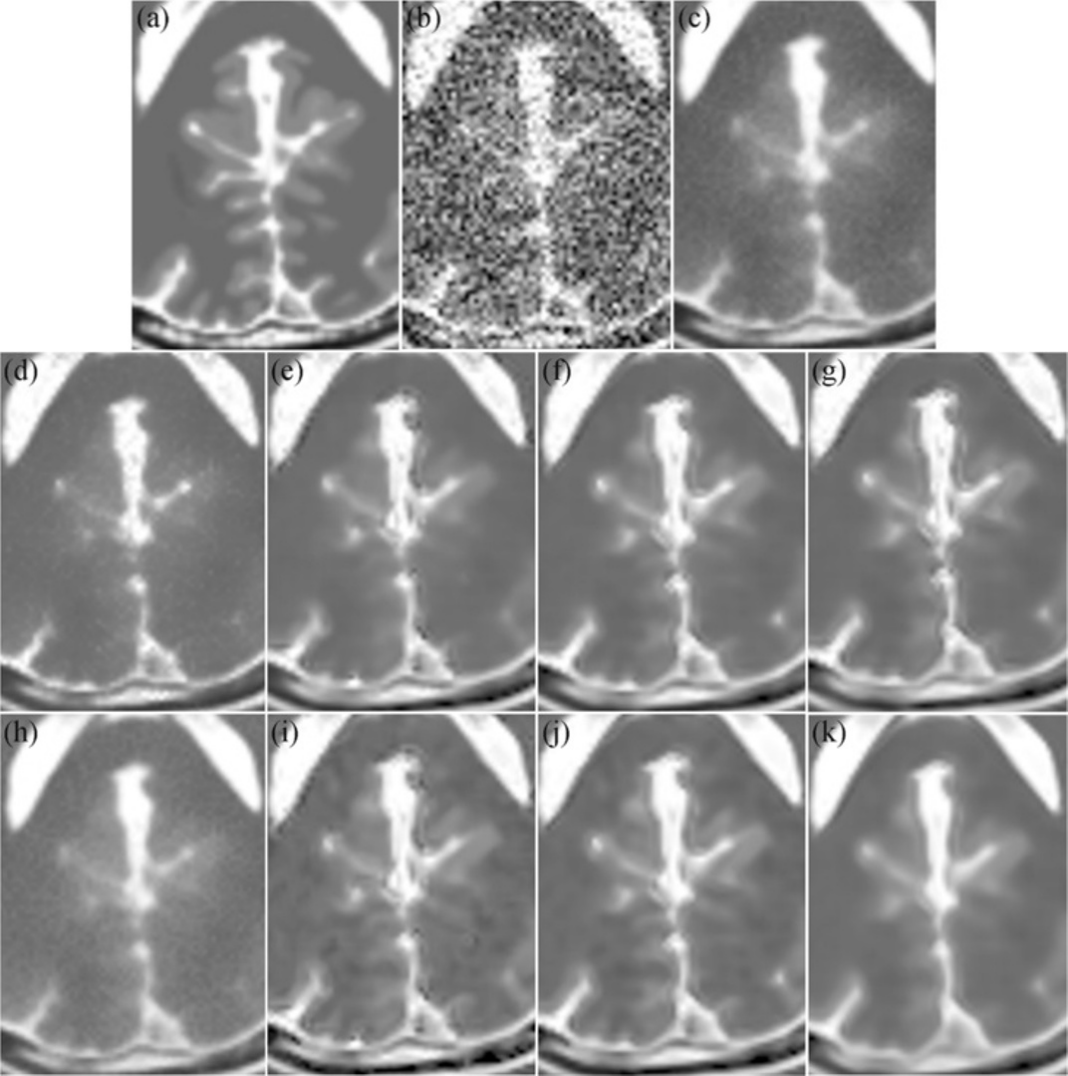
**Comparison of experiment results on T2-weighted MRI. (a)** original MRI, **(b)** noisy MRI with 17% Rician noise, **(c)** results of the original NLM filter, **(d)** results of the UNLM with squared magnitude transformation, **(e)** results of the PSNLM1 with median filter, **(f)** results of the PSNLM1 with Gaussian filter, **(g)** results of the PSNLM1 with anisotropic filter, **(h)** results of the UNLM with VST, **(i)** results of the PSNLM2 with median filter, **(j)** results of the PSNLM2 with Gaussian filter, **(k)** results of the PSNLM2 with anisotropic filter.

**Figure 17:**
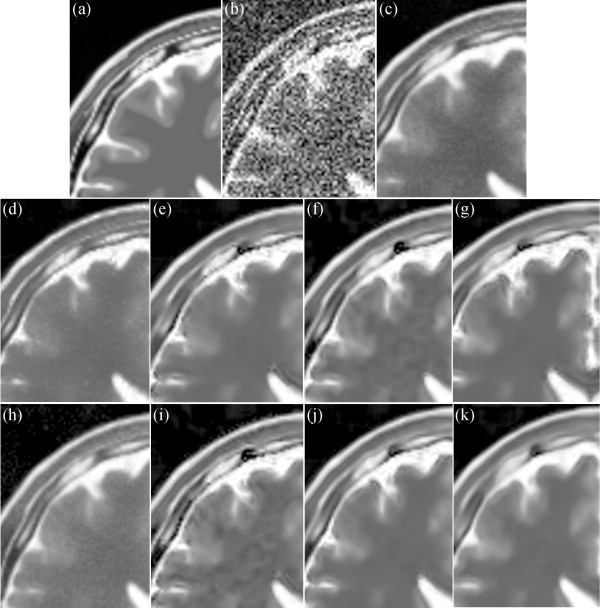
**Comparison of experiment results on T2-weighted MRI. (a)** original MRI, **(b)** noisy MRI with 17% Rician noise, **(c)** results of the original NLM filter, **(d)** results of the UNLM with squared magnitude transformation, **(e)** results of the PSNLM1 with median filter, **(f)** results of the PSNLM1 with Gaussian filter, **(g)** results of the PSNLM1 with anisotropic filter, **(h)** results of the UNLM with VST, **(i)** results of the PSNLM2 with median filter, **(j)** results of the PSNLM2 with Gaussian filter, **(k)** results of the PSNLM2 with anisotropic filter.

Figure 16 presents the results on the T2-weighted MRI image with 17% Rician noise. Figure 16(a) shows an enlarged region of the original MRI image, and Figure 16(b) shows the noisy MRI image. Figure 10(c) presents the result of the original NLM. Figure 16(d) and (h) present the results of the UNLM with the squared magnitude and VST, respectively. Figure 16(e) to (g) present the results of the PSNLM1 with median, Gaussian, and anisotropic filters, respectively. Figure 16(i) to (k) present the results of the PSNLM2 with median, Gaussian, and anisotropic filters, respectively. The result presented in Figure 16(c) exhibits the worst performance, and also, considerable noise occurs in Figure 16(d) and (h). Serious artifacts are observed in Figure 16(e),(i) and (g). Figure 16(f) and (k) are more blur than Figure 16(j). Figure 17 shows another enlarged region of the experiment results on the T2-weighted MRI image. The results are similar to those in Figure 16. And we can confirm that Figure 17(j) is the best.

### Real patient data

The brains of AD patients are atrophic, particularly at the hippocampus and the parahippocampal gyrus. Atrophy in these regions can be used as an early biomarker of AD [28]. To test atrophy degree in the brain, all AD and NC data are first denoised and then normalized to the Automated Anatomical Labeling template [29], which is a digital human brain atlas with labeled regions. These regions indicate brain structures. Given that the Jacobian map of the deformation field includes information on the local properties of the deformation field that can be used to detect volume changes, Jacobian logarithms are calculated such that the expansion and compression of the brain are scaled equally [30]. Log-Jacobians are calculated at each voxel in the volume to produce a map of the local expansion and compression of the brain. The Jacobian maps are smoothed by using smoothing kernels that measure 6 mm full-width at half-maximum for each of the 40 samples. By using SPM software [23], paired t-test is performed on the Jacobian maps of the AD and NC groups. An equal p value (p = 0.05) is used to compare the t-score obtained by the different denoising methods. The ratios of the detected atrophy region to the region of the hippocampus and the parahippocampal gyrus are listed in Table 2. The minimum ratio is 9.93, which is obtained from the original image. The maximum ratio is 58.69, which is obtained from the image denoised by the PSNLM2 with a Gaussian filter. The ratio of image denoising by the NLM filter is 55.98, which is only larger than that of the original image. This result indicates that image denoising significantly increases the ratios of the detected atrophy region to the region of the hippocampus and the parahippocampal gyrus. The transformation of Rician noise into additive noise can further increase the ratios. The PSNLM2 with a Gaussian filter is the best method for detecting atrophy regions.

**Table 2:** **The ratios of the detecting atrophy region and the region of hippocampus and parahippocampal gyrus**

1	2	3	4	5	6	7	8	9	10
9.93	55.98	57.66	58.55	56.03	57.74	57.52	57.09	58.69	57.76

1. Original image.

2. Image denoising by the nlm.

3. Image denoising by the unlm with squared magnitude transformation.

4. Image denoising by the unlm with VST.

5. Image denoising by the PSNLM1 with anisotropic filter.

6. Image denoising by the PSNLM1 with gaussian filter.

7. Image denoising by the PSNLM1 with median filter.

8. Image denoising by the PSNLM2 with anisotropic filter.

9. Image denoising by the PSNLM2 with gaussian filter.

10. Image denoising by the PSNLM2 with median filter.

Moreover, the proposed method is compared with other recently developed methods, including Rician image denoising, linear minimum mean square error (LMMSE) estimator [31] and total variation minimization (TV) [32]. A series of 2D T1 weighted MRI knee images are utilized to illustrate the performance of the proposed method. The image size is 383×420. Table 3 lists the results of the IMMSE, TV and PSNLM2, it can be seen that the noise level of the Rician noise is from 2% to 20%. While the proposed PSNLM2 method has the largest PSNR at each level of noise.

**Table 3:** **The PSNR of noisy image, results of LMMSE, TV and PSNLM2**

	2%	4%	6%	8%	10%	12%	14%	16%	18%	20%
Noisy	33.33	27.48	24.19	21.86	20.64	19.42	18.74	17.86	17.56	16.91
LMMSE	36.38	32.41	30.25	28.32	27.01	25.48	25.38	24.45	23.16	22.34
TV	36.87	33.19	30.30	28.53	27.21	25.63	25.18	24.69	23.36	22.40
PSNLM2	**37.60**	**33.93**	**31.78**	**30.12**	**29.14**	**27.93**	**26.92**	**26.13**	**25.34**	**24.52**

The larger PSNR indicate the better performance. The maximun PSNR is listed by symbol bold at each noise level.

## Conclusion

In this study, a PSNLM frame combined with image transformation is proposed for MRI denoising. After the Rician noise in MRI is transformed to the additive noise, the NLM filter is applied to the transformed image with weights computed from the pre-smooth image. The final denoising image is obtained from the inverse transformation of the NLM denoising result. Experiments are performed on the simulated data of the T1-, T2-, and PD-weighted MRI images that are downloaded from the BrainWeb database and real data (T1-weighted MRI images) downloaded from ADNI. Many traditional filters are tested as pre-smooth filters. In this paper, only the results using Gaussian, median, and anisotropic filters are presented because others such as trilateral filter and wavelet filter produce worse results. The proposed method is automatic and robust since it improves the denoising results greatly in all the experiments. One challenge problem for the proposed method is that it is comparably time consuming, which costs about 15 minutes on the image size 181×217×181 with 10 threads on the Dell server. The reason is that the image transformation of the NLM takes heavy computation. Recently, many works have been proposed with respect to decrease the computation time of the NLM, such as the FFT transformation [33], which is about fifty times faster than the original non-local algorithm and can reach the clinic application. The experiment results indicate that using the Gaussian pre-smoothing filter and VST produces the best results for the peak signal-to-noise ratio (PSNR) and atropy detection.
